# Editorial Notes, News and Miscellany

**Published:** 1914-02

**Authors:** 


### Editorial Notes, News and Miscellany.

THERE WERE ONLY TWO CASES of typhoid fever in the United States army last year. (Official.)

---

DO YOU "OPERATE" a case, or operate *on* one? I want to know. I observe that some of our best surgeons, in reporting cases, say they "operated" them.

---

DR. J. B. MCKNIGHT, of Brady, Texas, has been appointed physician in charge of the State Tuberculosis Sanitarium at Carlsbad, Texas, vice Dr. Bascom Lynn, resigned.

SENT FREE.—The Abbott Alkaloidal Co. will send free on request a copy of their new therapeutic price list, containing much valuable information. Flexible leather cover, 400 pages.

---

IMMIGRATION.—In the past ten years the number of aliens arriving on our shores was 9,856,000. Bahrenburg. We are already a race of mongrels. What will we be in fifty years?

---

"Every issue of your journal is a clarion note for the new age. Somebody hears it; somebody is getting better for the sound and thought, and that is what we all ought to do."—T. D. Crothers, M. D. [Hartford University].

---

THE WISE COUNTY MEDICAL SOCIETY now issues a bulletin. Dr. J. A. Embry is President of the Society, and Dr. L. H. Reeves is Secretary. The first issue is quite newsy. May tall oaks from little acorns grow. We commend their pluck.

---

CAN'T DO WITHOUT IT.—"The best medical journal in the world."—Dr. W. B. Mackey, Fort Worth, Texas.

"The very best journal extant."—Dr. R. F. Harnesberger, Beckville, Texas.

"It is the best journal that comes to me."—Dr. W. R. Blackshear, Chireno, Texas.

["And so say we all."]

---

REPORT OF THE NATIONAL CONFERENCE ON RACE BETTERMENT.

BATTLE CREEK, MICHIGAN, January 30, 1914.

*Editor Texas Medical Journal:*

Four hundred men and women of prominence, comprising the first representative group of scientific experts ever gathered in America for that purpose, met in Battle Creek, January 8-12, to assemble evidence of race deterioration and to consider methods of checking the downward trend of mankind. The meeting was known as the First National Conference on Race Betterment. Through the co-operation of the press, the objects and aims of the Conference have been very widely disseminated and a resultant influence for better race ideals is anticipated.

Already, the effect of the Conference is apparent in Battle Creek where popular interest in mental and physical efficiency was awakened by a series of public school tests, which showed an alarming percentage of defective children in all grades.

The Conference had its inception in the efforts of four men, particularly interested in race betterment—Rev. Newell Dwight Hillis, Pastor of Plymouth Chourch, Brooklyn, New York; Dr. J. H. Kellogg, of the Battle Creek Sanitarium; Sir Horace Plunkett, former Minister of Agriculture for Ireland, and Prof. Irving Fisher, of Yale University. At the invitation of a central committee chosen largely by these men, fifty men and women of national prominence in the fields of science and education consented to share in the program. Their addresses, together with open discussion of many of the points considered, constituted a very widespread study of all phases of evident race degeneracy and the advocacy of many ideas of reform. Some of the suggested methods of improvement are frequent medical examination of the well, outdoor life, temperance in diet, biologic habits of living, open-air schools and playgrounds, the encouragement of rural life, the segregation or sterilization of defectives, the encouragement of eugenic marriages by requiring medical certificates before granting license and the establishing of a eugenics registry for the development of a race of human thoroughbreds.

Among those having a share in the program were: Rev. Newell Dwight Hillis, Jacob Riis, Judge Ben B. Lindsey, Booker T. Washington, Dr. Victor C. Vaughan, Dr. S. Adolphus Knopf, Dr. C. B. Davenport, Dr. J. N. Hurty, the Very Reverend (Dean) Walter Taylor Sumner, and many others of equal prominence.

Some of the interesting statements of the Conference are summarized as follows:

"It will be no easy task to improve the race to the point where there will be no dependent children, but the elimination of the dependent child will be one of the best indices of the superiority of our national stock."—Dr. Gertrude E. Hall, New York State Board of Charities.

"I believe that a great deal can be done by publication of facts as to the physiological effects of alcohol, in the way of inducing educated and intelligent people to conserve their health by limiting the use of alcohol or giving it up altogether.—Henry Smith Williams, Author.

"Eugenics does not eliminate romance. We eugenists believe romance should be retained. Through the past, it has proved a good thing."—Prof. Roswell H. Johnson, University of Pittsburg.

"In order that the race may survive, it will apparently be necessary to make a eugenic selection of healthy mothers, and to provide that the cost of bearing and rearing children shall be equally shared by all."—Prof. J. McKeen Cattell, Editor *Popular Science Monthly*.

"The boys are learning that they have a calling just as sacred as the call to motherhood, and that is the call to fatherhood."—The Very Reverend (Dean) Walter Taylor Sumner, of Chicago.

"The negro in the South, with all his weaknesses and handicaps, is not yet, in any large measure, in the ditch."—Booker T. Washington, Principal of Tuskegee Institute.

"We must cultivate pure blood, instead of blue blood, if we would develop a race of human thoroughbreds."—Dr. J. H. Kellogg, Superintendent Battle Creek Sanitarium.

